# The Duty of the Dentist to His Profession, His Patient and to Himself

**Published:** 1866-03

**Authors:** Julius Chesebrough


### THE DUTY OF THE DENTIST TO HIS PROFESSION, HIS PATIENT, AND TO HIMSELF

BY JULIUS CHESEBROUGH.

[Read before the Michigan Dental Association.]

Whatever may be said upon this topic, on one thing we can certainly rely—that we all fall far short of our duty. To know how to perform that duty, so as to do justice to all, is worthy of our study and care. God has ordained for us a work, and we have chosen this one, upon which to hang our attainments, to raise them, each day, higher upon the standard of excellence. Let us look into the sphere we are filling, and find out, if we can, what the fulfillment of God's ordinance is.

I say we have chosen this work, and to labor in this field, for our life-task,—a field immeasurable, unbounded, unfathomable. He has endowed us with certain faculties, which we are not to hide in a napkin, but to improve, and add interest, so that when we surrender them, they will be greatly increased. He has endowed us with a brain, a seat of reason, upon which impressions can be indelibly printed, and from which emanate thoughts of good or evil. He has given us eyes that we may see, ears that we may hear, and an utterance that we may be able to impart what we know. All these are *not for our own good*, but for the benefit of mankind. His intention never was that we should be for ourselves alone, but that we should minister to the wants of our neighbors as well as to our own.

Were it best that there should be no divinity, no growth in mind, no continual approach toward perfection, He would never have "formed us after his own image." But that He has done so, and that the race of man comprises so many beings, is proof that His will is that we go onward and upward.

In consideration that our duty is one of progress, do we fill the measure God has ordained for us; and if not, wherein do we fail?

I call your attention to these three topics:

Our duty to our profession, our patients, and ourselves. I speak to you, individually, and, classing as a whole, I embody the question into that word, *Our* duty,—not yours, individually, nor mine; but let each one of us take these matters to himself, and ask, Have we done our duty?

The question may arise in the mind of some one here, what duty do I owe to any of these three that I do not fulfill?

Let us look upon our duty and see what it really is.

For long, dark, weary years, our profession groped its way, "dragging its slow length along," until it has taken a sudden start, and has now its place recognized and accepted by the world.

From whom did it receive its impetus, and what were the labors of those that gave it that impetus? We all know whom we honor, when we proudly repeat the names of Hunter and Bell, Tomes, Kolliker, Townsend and Harris, and the goodly number of the early Apostles, who dispensed the doctrines that we all know, little by little. Have we done our duty to our predecessors, who have given us of their toils and labors, years of night-study and investigation? Let each one of us answer the question, and answer it well; and from this moment, henceforth, go and do likewise.

Our profession, I have said, has received an impetus unparalleled in any record. It has sprung into existence during the past century, and has now an accepted position from which it will never recede.

But let me ask each one of you, how much have you helped to advance it during your lifetime; and where have your labors shown themselves? Would to God that I had the whole world of dentists to put this question to, and to hear the answer that must come up from some breasts. Have your labors been such as to elevate the profession above that of mere mechanical employment? Have your researches into that field of science, ready for the sickle of investigation, been such as to enable you to bind up a sheaf, from which you may separate the grains of fact from the straws of theory, and bring those grains before the feet of your compeers, and say, "here is my contribution? Or have you been content to look in upon the workers in the field, and see how they gather in the straws, and in the great showing, been content to look at their grains, and gain from their labors, that for which you have returned no equivalent? Have you, by your example and counsel, lifted up your ignorant brother worker, so that he will look upon you as a friend of elevation? Or have you, by derogatory remarks, tended to degrade, rather than elevate? Have you studied day and night to fit yourself for the sphere you occupy? Have you investigated the science of disease, so that you may know how to combat it in the thousand shapes that it manifests itself upon those organs, which it is your specialty to treat? Have you made a note of such labors, and given them to the world, thereby adding your grain to the store already gathered? Or, on the other hand, have you been content to set yourself down, in indifference and carelessness, and eat of the fruit of others' labors? Have you given to your patients that high character of operations, which approach perfection, thereby enabling them to pay regard to these organs which are to them worth more than jewels, and which brings the profession up, in their minds, to the standard of excellence, and wrestles it from its low position where, through carelessness of operators and ignorance, they have consigned it?

You may say, as I have heard some say, "I can not do such things in my practice, my patients wont pay for it." I say *you can* do such things in your practice. Do you *always* look merely to the pay for the work accomplished? Then, indeed, you will always be a dolt. Strike above all this,—let such thoughts take themselves to the wind; and strive to bring up your profession, instead of bearing it down. I tell you, sirs, your patients are able to discriminate; and no one will ever object to remunerating for benefit received.

It may take you some little time to educate your patients, but they will be easily educated; and once educated to appreciate, is a faculty never lost.

How much better for you to feel that a patient owes you much beside the money consideration received, for the service done, than to meet one and have your conscience tell you you did not do that patient justice; for his inability to pay, or your ignorance of the case, has rendered him a deformity. I always want my patients to feel that they still owe a debt of gratitude for service done.

We need more careful, earnest thinkers, more hard investigators, more workers who will record their research. We have no literature, or next to none. What we have is good enough; but it is not enough. Your daily practice affords something that is of moment to the rest of the profession; then why not give it?

A young man once said to me, "Your profession seems to comprise so little that it must be very easy." Years have passed since that day; and his opinions have wonderfully changed. "I thought," says he, now, "that the practice of dentistry was that of filling, extracting, and inserting teeth; but now, after years of study, I find that I have always been in error. I have found out that he who only does these three, without inquiry as to the nature and source of disease, knows nothing. I thought it an easy road at first; but I find it a rough one, and that I have to pick my way among its rocks and stumps, and in the darkness, that sometimes lies before me, I find myself lost." And so will we all be lost unless we study our charts, and wherever we find a sunken rock, note it, at once, for the benefit of humanity. What would the world say of that sailor that discovers a reef in mid-ocean, and say, "I will say nothing of this to any one. I will know where it is, and it will not baffle me more. Let those that sail this way find it out; and what matters it to me, though a shipwreck ensues, and life is lost?" Would he not sink into utter disgrace; and would not he receive the contempt of the whole world? And how do you—one and all of *us*—differ from him?

Your duty is, by your industry and faithfulness, to elevate your profession,—by your daily walk and conversation to give it additional dignity.

And what to your patient?

Frequently the very bliss of ignorance presents itself to you. What is your duty in such a case? to let that ignorance remain? or weed it out from a brain adapted for higher things? The preconceived idea that many have received is, that their teeth are worthless as soon as they give them trouble; and the sooner they are rid of them the better. Have we not a duty to perform in such a case as this? and must we not, at once, turn teacher, until we see that our patient has learned the first rudiments of common sense? We are all very susceptible of fact, and are easily convinced. Let us but know the fact, and we are at home. And our patients are as easily influenced as ourselves. We have but to speak the word, and our knowledge of the matter is at once accepted.

We must point out the error of their lives, and let them understand that these organs are of more value than any others. Show them that, by proper treatment, they can be saved; and you will not have to teach those patients again. I can call to mind many who are among my best patients, who are educated patients, who doubted greatly at first, but who are now beyond a doubt.

Our duty is to give, as freely as we have received, of that which will tend to elevate our calling. Indulge in no theory, but confine yourself always to the fact; and give that fact as tersely as words will allow, coming down, for the time being, to the level of the capacity of your patients, and let them ascend, step by step, gaining confidence with you, at each successive one, until they will accept your direction as law, and abide strictly by it. Never deceive them upon the lightest matter or most trifling occasion. Let them gain confidence in you, and your work is done. Where ignorance is at fault, educate it to the excellent standard; where obstinacy comes in contact with you, send it away, have nothing to do with it, wash your hands of all connection with it, and let it depart in peace. Education will always appreciate a well appointed labor; but stubbornness—never. Send it out of your sight. When a patient comes to your hands for treatment and advice, you, must be prepared *to give* that advice; and woe to you, in the last day, if you have given it wrong. God's judgment is everlasting, and will surely find you out, when all things are to be known. It will not be your fault if, after knowing what is right, and knowing, dare maintain, your patient does not accept your advice. But do not be drawn into doing a wrong act because your patient insists upon it. Have the manliness to come out boldly and say, "*I wont*," even though the heavens fall. Always advise your patient to have the best operation performed, regardless of expense. No one can ever master his profession that will advise otherwise. Your patrons will, sooner or later, find you out; and your sayings and doings will surely come home to roost.

Our duty is to be always kind and gentle—never rash or petulant. No annoyance should ever rouse our temper; but we must smother the burning flame and quench the fire. Your patients will soon become affable and easy if they see you undisturbed. But when you are cross, and speak sharply, they will ever remember it.

If you have an operation of magnitude and severity, acquaint them of the amount at once. They will abide by your decision better than when deceived. Whenever pain can be mitigated, it must be done. We were not ordained to create pain, but relieve suffering; and this we can do, if we but know our duty.

With children, our duty is a greater one, — kindness, all over, within and without, in the very atmosphere, in all the surroundings, must be inflexibly maintained. By so ingratiating ourselves into their affections, we may make our operations a source of pleasure instead of dread. We hold the power within us; and, if we will, we can do as we please. We must be firm, yet courteous; inflexible of purpose, yet affable of manner; and, knowing our duty, maintain it.

Many with whom I have spoken these things have said to me, "I must live; and to live, I must cater to the fancy of my patient." To all such I can only say, that if, through your own or your patient's ignorance, you cater to their fancy, you do yourself and them great wrong, and the gain that results from such action, affords no pleasure. Better starve than sin outright. Be poor, but do your duty; for gold, gotten through deceit or falsity, will melt away.

And what of the duty you owe to yourself. This age demands intelligence; it demands thought; it demands knowledge; it demands *brains*. Are we up to the standard? Do we appreciate our position? Are you, gentlemen, each one of you, satisfied with your attainments? Are you doing your duty to yourself, with the knowledge you have, and would you trust yourself under your own hands? Are we thoroughly acquainted with the intricacies of disease, and the complications of the parts which we have to treat? Can we grapple with all the minutiæ of morbidity of function, which every day comes under our observation?

How many pass over these things as trifles, which are "apples of gold." I have heard many persons say, "I never think of examining into the condition of the secretions of the mouth, or of any thing but the teeth. I look at them and what I can not fill I pull. I never ask about the patient's general health, symptoms or afflictions. I do what I can to the teeth, and let them go." I tell you, sir, that if you did inquire about these minutiæ, and study more of the diseases that have their origin or influence in or upon the teeth, your labors would be much easier than they now are, and your patient would be better satisfied. We must look these matters *square in the face.* Our profession is not even with the age, it is far behind it. There are some who are equal to the hour; but the laborers are few. The age is demanding of us better work, more perfect operations, and, above all, a knowledge of disease and complications that show themselves upon the teeth, in order that we may save from ruin the thousands upon thousands of organs that now go to destruction.

It is no less a duty to the profession and our patients than to ourselves that we should know these things. Our education must be of a higher order than that of being self-taught, or of receiving it from a self-taught preceptor. The world demands it, our patients need it, the profession will insist upon it.

Do we, as a profession, study enough? I emphatically say no!

Do we think enough? No! Do we investigate enough? No! Do we write enough? No!

From whom do we receive all our intelligence? From a score of dental writers; hardly more.

It is a burning shame to us, that the labors are thrown upon the shoulders of these few, who need the time that they give for our benefit, for their own rest.

Let me put this question right home to each one of you, sirs, Have you done your duty in this respect? Freely ye have received; have ye as freely given?

It is of no use to say you can not write. Can you think, or act, or do, or say? Then say it at once. I can not learn for you; but I can learn something from you. By interchanging our ideas, we get them sharpened, and what's the harm if they do cut close? So much the better.

Interchange ideas with your brother practitioner; discuss the thousand topics (every one of which is almost an untrodden field) whenever you have opportunity. It will give you an insight such as you can not gain otherwise. And your neighbor and friend (for what are we other than friends) may know something that you do not, as the result of his investigations, and you, in turn, impart to him that something which he may desire to know.

We are none of us too old to learn; and I never meet a professional man, but that I talk with him; and I always feel better for it.

We must study more than we do. Work by day, study by night; and by having a system, and devoting a few hours every day, we will be astonished to know how much we will achieve in a single year.

We must do our duty above all else, contribute our share to the general fund of knowledge. What if every one were silent? Where would our knowledge be? and of what avail would our profession be?

What sort of workers would we be were we closed up within ourselves?

Let the ball roll onward, gathering as it goes; and let us urge ourselves to new endeavors in the field of dental science. Look at the immensity of the sphere that has not been touched upon by any writer. Scarcely any subject has had more than a passing thought, compared with the great unwritten, unsaid. How little has the pathology or physiology of dental science received from pen of the dental writer, and how little has surgery received, where there is so much that is of great importance!

Who, among us, has not had strange cases come under our hands, and from which we could write an instructive article.

And when we meet together to exchange our views, do we come prepared to instruct as well as to receive instruction?

There is not one of us that could not bring something to instruct the whole. But have we done so?

If not, then let us start out on a new track, and make a resolve, that, from this time forward, we will be what the age demands. We will learn what is now unlearned. We will do what we have left undone. Where we have failed, we will now succeed, and where have destroyed, we will now build up. We will strive to have our operations perfect and successful, and, by study, will be enabled to combat disease, and devote a part of our time to record our labors for future generations. My watchword shall be *I will;* and I will ever remember that "what man has done, man can do."

With such a resolve, well made, and surely kept, we will grow apace. We may be the beacon light to guide some follower over a troublesome passage. We may be benefactors to the human family, and do our duty to the Profession, our Patients, and to Ourselves.

